# A Ten Years' Review of the Expenditure of the London Hospitals and Its Lessons

**Published:** 1905-02-04

**Authors:** 


### A TEN YEARS' REVIEW OF THE EXPENDITURE OF THE LONDON HOSPITALS AND ITS LESSONS.

A decade ago it was impossible to draw any accurate conclusions from a study of the finances of the London hospitals. Owing to the diverse methods of account-keeping employed, and to the inefficiency in some cases of the system adopted, the hospitals were deprived of the stimulating force of fair comparisons, and the public was denied the privilege of knowing with certainty in what manner their contributions were being expended. Everyone is aware of the necessity for proper statistical returns in the successful conduct of any enterprise, and the adoption throughout the voluntary London hospitals of the uniform system of accounts supplied what was at the time probably the most pressing need in the management and control of these institutions. That system has now been in force sufficiently long to render possible a critical review of the cost of administering medical relief under the existing arrangements year by year in the metropolis. As would naturally be expected, a study of the figures is pregnant with suggestions for modifications, and even for reforms of the present methods of hospital management. In the 1905 edition of "Burdett's Hospitals and Charities," which has just been issued, a number of tables is given, showing at a glance the income and expenditure for the last ten years of the voluntary hospitals, together with the numbers of patients treated within their walls; and every thoughtful citizen would do well to ponder on the conditions which these tables reveal, and to give himself up for a while to the reflections which will naturally arise. Table A, on page 58, gives the income and expenditure of the 94 voluntary hospitals in the County of London during the 10 years 1894 to 1903 and shows that the total amount of money contributed to the London hospitals in 1903 was £1,331,090, as compared with £807,785 in 1894, an increase in the decade of nearly 65 per cent. This noble response on the part of the charitable public to the urgent appeals made to them has unfortunately not found its equivalent on the side of hospital management. Year by year the expenditure has grown; in 1894 the total was £837,725; in 1903 it had risen to £1,341,641. Now, this increase cannot be attributed to the increase of the population of London. Reliable statistics show the increase of population during the ten years under consideration to be about 7¼ per cent., the beds which have been added during the same period amount also to 7¼ per cent., but the number of patients treated has increased by 23 per cent., so that, although it was considered in 1894 that the necessities of the Metropolis were more than met, it appears that the hospitals have voluntarily added to their obligations three times as much work as the natural increase in the population would have put upon them, and this in face of the great efforts which were being made on their behalf by the benevolent public. It is manifest from these figures that reforms are desirable in two main directions, namely, the careful control of expenditure in every branch, and the limitation of free medical relief to those for whom the charitable subscriptions are asked and received. The growth of socialism which is such a salient feature of to-day may perhaps carry the promise of a better future for mankind, but there can be no doubt that certain of its tendencies call for the strenuous opposition of every robust member of the community, and one such tendency is the increasing readiness with which individuals are prepared to accept charitable assistance. A sturdy independence among its people is a matter of supreme importance to the vitality of the nation, and in so far as hospital abuse tends to undermine this independence, the question becomes one of national importance. The necessity for careful control of expenditure needs no emphasis; it is self-apparent.

In a letter appearing in the *Times* of Wednesday Sir Henry Burdett enters fully into the whole question involved, and insists on the necessity for cutting down the admission to voluntary hospitals of free cases by forcing the majority to obtain medical relief in the ordinary way; and he further urges the desirability of hospital managers bringing the accommodation they afford to sick people up to the requirements of modern times by opening pay wards under conditions which will be adequate to protect and meet the reasonable claims of all the interests concerned. It may be hoped that the wide publicity of these views which the columns of the *Times* will afford may lead in the near future to the reforms which to-day are so much needed in the interests alike of the hospitals and of the general community.

Mr. Henry W. Marshall of the Stock Exchange has made an offer to King Edward's Hospital Fund of certain freehold land in London on condition that £18,000 is subscribed to the permanent income fund before July 31. The property consists of business premises and is let to one tenant on a repairing lease, of which 25 years are unexpired, at the rental of £420 per annum. It is believed to be worth £12,000.

